# The inductive effect of musical mode types on emotions and physiological responses: Chinese pentatonic scales versus Western major/minor modes

**DOI:** 10.3389/fpsyg.2024.1414014

**Published:** 2024-06-19

**Authors:** Yihe Jiang, Maoping Zheng

**Affiliations:** ^1^Key Laboratory of Cognition and Personality (Ministry of Education), Southwest University, Chongqing, China; ^2^School of Psychology, Southwest University, Chongqing, China; ^3^School of Music, Southwest University, Chongqing, China

**Keywords:** Chinese traditional pentatonic modes, emotional experience, Western major and minor modes, skin conductance, skin temperature, heart rate

## Abstract

**Background:**

The value of music lies in its ability to evoke emotions. People can gain emotional experiences in music and can also regulate their own emotions through music. Music has its own structural rules, and exploring the relationship between musical structure and emotions is an important approach to understanding the mechanism of music-induced emotions. Musical mode refers to the arrangement of intervals around the tonic, presenting different musical modes based on the central tone and the arrangement of intervals, including Chinese pentatonic modes and Western major and minor modes. Musical morphology indicates significant differences in the construction intensity of traditional Chinese pentatonic modes and major and minor modes, affecting their mode forms and thus determining their adaptability to external influences.

**Aims:**

Exploring the modalities of music and the effects of individual music training experiences on emotion induction; validating whether musical modes exhibit cross-cultural universality in the process of emotion induction.

**Method:**

This study recruited 65 university students as participants (34 with music training experience, 31 without music training experience). Through a passive listening paradigm using the GEMS and combined with a biofeedback equipment, it explored the differences in behavioral and physiological indicators (skin conductance, temperature, heart rate) of emotional experiences (basic and aesthetic emotions) influenced by the modal forms of Chinese traditional pentatonic modes and Western major and minor modes.

**Results:**

Firstly, the arousal level of music emotion is a primary factor influencing individuals’ aesthetic emotional experiences in music, which is related to the intensity of modal construction in music; Secondly, the emotional pleasure and skin temperature change induced by pentatonic music are greater than those induced by major and minor modes; Thirdly, the arousal level, electrodermal change, and heart rate variability of major and minor modes are greater than those of pentatonic music; Finally, music training experience enhances college students’ familiarity and preference for pentatonic music, thereby strengthening the electrodermal physiological indicators of emotional experiences.

**Conclusion:**

The different modal forms of music express different levels of emotional arousal, leading to differences in individuals’ emotional dimensions and physiological indicators in music. Additionally, individuals’ music training experiences and cultural backgrounds also influence their experience of music emotions.

## 1 Introduction

Music can elicit emotional responses in individuals (Scherer and Zentner, 2008). People not only perceive and experience the subtle emotions conveyed by music but also can change their own emotions and relieve stress through listening to music. From a musical perspective, music has the ability to express and induce emotions, while listeners are able to perceive and experience the emotions conveyed and evoked by the music (Schubert, 2013). The emotions expressed in music correlate with those perceived by listeners, while the emotions induced by music correspond to those experienced by listeners. Music emotion perception, refers to the audience’s awareness and judgment of the implicit emotions in a musical piece without necessarily basing it on their own feeling. Music emotion experience refers to the subjective emotional feelings induced by music stimuli, accompanied by physiological reactions and behavioral tendencies, such as physiological arousal and facial expressions (Hallam et al., 2009; Juslin and Sloboda, 2013). Furthermore, as a form of social and cultural expression, music reflects an individual’s socio-cognitive level through their perception of music-induced emotions (Allgood and Heaton, 2015; Trehub et al., 2015). Music, as a cultural symbol, possesses its own formal structural rules. The theory of cultural specificity suggests that due to differences in musical cultures, each culture will have its own unique music composition techniques, leading to differentiation in the forms of musical works (Livingstone and Thompson, 2009; Higgins, 2012). This means that different musical cultures exhibit differences in the way emotions are expressed through structural characteristics and organizational rules of music. However, some previous cross-cultural studies have suggested that there is a certain universality in the expression of emotions through music, primarily manifested in basic emotions (Balkwill and Thompson, 1999). Whether this result can be extended to the perception and experience of complex emotions requires further investigation. Combining the emotional experience of music with music therapy can effectively aid patients with mental disorders (Konieczna-Nowak and Trzęsiok, 2018). Music therapists can evoke patients’ knowledge, experiences, and preferences by selecting appropriate music, guiding them to fully engage in the aesthetic experience of music, thereby effectively alleviating the clinical symptoms of patients with mental disorders (Silverman et al., 2016). By exploring the influence of different musical forms on emotional experiences across different cultural backgrounds, we can deepen our understanding of how different cultures comprehend and express emotions through music, uncover the impact of cultural differences on music emotional experiences, discover universal emotional characteristics, as well as cultural-specific expressions of emotions, thereby enriching and refining the theoretical framework of music psychology, and promoting the application of music in health and medical fields.

**Figure 1:**
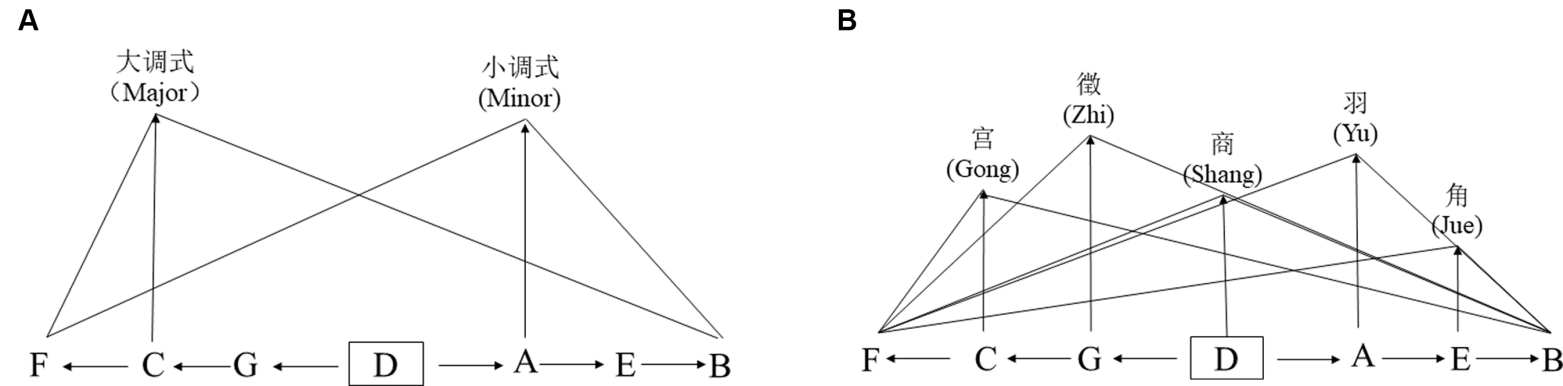
Construction strength diagram of major and minor modes and pentatonic scale based on the circle of fifths (Source: Pu,Hengjian, 2020). **(A)** major and minor modes; **(B)** pentatonic scale.

Musical modes involve organizing seemingly unrelated tones according to specific interval relationships, reflecting differences in the stability of scales within the tone series. Western music, based on the tonic and the arrangement of intervals, has developed major and minor modes (Bai et al., 2016); in contrast, the traditional Chinese pentatonic scale features a less dominant tonic, allowing any of the tones to potentially serve as the main tone, thus constituting the pentatonic scale (Pu, 2020) Table 1 illustrates the differences between the traditional Chinese pentatonic scale and the Western major-minor scale. The major and minor modes and the pentatonic scale show different tensions on the basis of the circle of fifths structure (see Figure 1), where the cumulative energy of the five modes in the pentatonic system equals the total of the two major and minor modes in Western music. Therefore, there are noticeable differences in the construction strength of modes, with the major and minor modes having a stronger construction strength and the pentatonic scale exhibiting weaker construction strength. In terms of melodic form, the modal logic of the Western major and minor system is clear, making the music rich in tension, with a stronger degree of stress and more intense emotional arousal; the traditional Chinese pentatonic scale, due to its weaker tonic control and vague modal logic, thus leads to a more gentle expression of musical emotions (Pu, 2020).

**Table 1:** Comparison of differences between Chinese traditional pentatonic scale and Western major/minor scale.

Feature	Chinese traditional pentatonic scale	Western major/minor scale
Scale structure	Pentatonic Scale: Gong, Shang, Jue, Zhi, Yu	Diatonic scale: C, D, E, F, G, A, B
Mode composition	Having pentatonic nature, the core consists of a triad	The foundation of the mode is a tetrachord
Timbral characteristics	Simple, Rustic, Concise	Complex, Rich, Exquisite
Formation reasons	In both academic and artistic realms, Chinese culture tends toward directness, pursuing epiphany and insight into essence. Its expression is typically concise and profound, emphasizing conveying rich connotations with minimal words, using simple strokes to create profound charm.	Western culture pursues details and precision, deriving complex logical systems, establishing extensive philosophical frameworks, accurately calculating minor errors, and forming rigorous and precise definitions.

Investigating the relationship between musical mode form and emotion processing is an important approach to understanding the underlying mechanisms of emotion induction in music (Gabrielsson and Lindström, 2010). The multiple mechanism model of music-induced emotions, proposed byJuslin and Västfjäll, (2008), known as the BRECVEM (A) model, has had a significant impact. This model comprises eight mechanisms: Brain stem reflex, Rhythmic entrainment, Evaluative conditioning, Contagion, Visual imagery, Episodic memory, Musical expectancy, Aesthetic judgment. The model explains the mechanism of music-induced emotions from an evolutionary perspective, based on distinct brain functions that have evolved over time to establish specific orders. Furthermore, due to its reliance on different brain functions, it exhibits unique functional characteristics (Juslin and Sloboda, 2013), which can be utilized to explain individual differences in emotion induction. That is, different listeners may activate distinct mechanisms when listening to the same piece of music, thus eliciting different music-induced emotional experiences (Juslin et al., 2022). The Clues-Consistency Model suggests that factors influencing the emotional experience of music include not only structural elements of the music itself (such as mode, rhythm, and pitch) but also presentation aspects of the musical piece (such as tempo, timbre, dynamics), individual traits of the listener (such as personality, gender, musical training experience), and situational factors during music listening (such as attending a concert) (Juslin, 2003, 2013; Juslin and Lindström, 2010), where the structural elements of the music itself and the presentation aspects of the musical piece serve as direct cues for inducing musical emotions. The ‘reciprocal feedback’ model is a music communication model that combines two parallel component models to explain the mutual influence relationship between music performance and audience response. Compared to the traditional transmission model or one-way influence model used in music emotion experience research, this model is a more comprehensive theoretical model that can better understand the complexity of music emotion experience. The model focuses on revealing the complex relationship between music performance and audience response, including the comprehensive impact of individual psychological characteristics, musical features, and contextual factors on emotional experience. It attempts to consider the role of multiple factors in music emotion experience and emphasizes the importance of their interactions (Hargreaves et al., 2005). The emotional experience of music is related to individual preferences and likes, Berlyne (1971) proposed the psychobiological theory of preference, which explains individual preferences for stimuli by emphasizing the relationship between cognitive and emotional responses to stimuli. According to Berlyne’s theory, individual preferences for music may be influenced by factors such as innovation, uniqueness, and complexity in music. When music presents an appropriate level of novelty and complexity, individuals may experience an “optimal level of arousal, “enhancing their preferences for the music and positive emotional experiences. Furthermore, according to Berlyne’s theory, music can also evoke a state of pleasurable tension in individuals. Factors such as emotional expression in music, changes in musical structure and dynamics, and the emotional content conveyed by music may elicit emotional responses and psychological tension in individuals. These emotional responses can influence individuals’ preferences for music and emotional experiences. In music, tonality is an important factor involving the pitch structure and interval relationships in music. There are significant differences in pitch structure and interval relationships between Eastern and Western music tonalities, which may have varying effects on individuals’ emotions. There is not much research on the relationship between music training experience and emotional experiences, in this study, we also explored the impact of music training on emotional experience. Previous research has indicated that participants with music training experience may engage in “analytical listening” rather than “emotional-experiential listening” during music listening processes, participants with music training experience tend to experience music emotions more intensely than those without music training experience (Lehmann, 1997).

The tonality of music is associated with emotional hues, major mode music sounds enthusiastic and bright, capable of eliciting positive emotions in individuals, whereas minor mode music sounds somber and plaintive, capable of eliciting negative emotions (Webster and Weir, 2005; Juslin and Lindström, 2010; Parncutt, 2014; Straehley and Loebach, 2014; Bai et al., 2016). Methods for measuring music-induced emotions often employ self-reporting and psychophysiological measurements. Self-reporting methods mainly include Likert scales, adjective checklists, and free reports. Early research on music emotional experiences was primarily based on traditional categorical methods of basic emotions (discrete emotion models or emotion dimension models). However, traditional methods of categorizing basic emotions cannot adequately describe complex emotions (Hunter et al., 2008). Therefore, researchers began exploring specific models of music, suggesting that aesthetic emotions differ from everyday emotions, as they arise in contexts unrelated to self-interest or goal-directed behavior (Scherer and Zentner, 2008; Liu et al., 2021). Zentner et al. (2008) collected thousands of participant samples at the Geneva Music Festival and developed the Geneva Emotional Music Scale (GEMS), proposing a nine-factor model: Wonder, Transcendence, Tenderness, Nostalgia, Peacefulness, Power, Joyful, Tension, and Sadness. As subjective self-reports cannot verify the physiological components of emotional induction (Eerola and Vuoskoski, 2013), some studies employ indicators such as skin conductance, skin temperature, and heart rate for detection, although there is currently no consistent research result. Previous studies have shown that skin temperature is related to the emotional valence of music, but research on the emotional induction of music tonality yields inconsistent results. Some studies suggest that skin temperature is higher for major keys than for minor keys (van der Zwaag et al., 2011), while others indicate higher skin temperature for minor keys (Lundqvist et al., 2009). Skin conductance is related to the arousal level of emotions (Keil et al., 2008; Kreibig, 2010; Levenson, 2014). Research shows that when listening to highly arousing music, skin conductance increases (Yamamoto et al., 2007), and (Lundqvist et al., 2009) found that major key music induces higher skin conductance. Heart rate is not related to the valence of stimuli but is an effective indicator of the arousal level of emotional experiences (Pfister et al., 2011). Additionally, some studies, from a cross-cultural perspective, suggest that Western major key music and Chinese traditional music’s Gong mode share similar emotional hues, as do minor key music and Yu mode music (Bai et al., 2016; Yang et al., 2022), (Bai et al., 2016) explored the emotional induction effects of Western and Chinese music using subjective assessments (emotional valence, arousal, and tension) and physiological indicators (skin conductance, finger pulse rate, and skin temperature). The results indicated that major key and Gong mode music induced positive emotions, while minor key and Yu mode music induced negative emotions; major key music induced higher skin temperature than minor key music, and Gong mode music induced higher skin temperature than Yu mode music. Zhang and Pan,(2017) used a multi-channel physiological recorder to investigate the role of tempo and tonality of Western and Chinese music in emotional responses. The experimental results showed that major key, fast-paced music had higher subjective ratings than slow-paced, minor key music; fast-paced, major key music exhibited higher heart rate and skin conductance indices than slow-paced, minor key music; and the skin temperature of major key music was higher than that of minor key music. Previous studies have often determined the emotional color relationships between the Gong and Yu modes in the pentatonic scale and the corresponding major and minor modes in Western music, followed by empirical research for validation. However, there has been a lack of exploration into the emotional colors of the Shang, Jue, and Zhi modes in pentatonic music. Therefore, in this study, we first validated the emotional colors of each mode in the pentatonic scale in the materials section, followed by the selection of experimental materials for comparison with major and minor modes.

This study adopts a passive listening paradigm, coupled with subjective assessments and physiological indicators, to investigate the emotional experience differences between college students with diverse musical training backgrounds regarding the Chinese traditional pentatonic scale and Western major and minor scales. We hypothesize as follows: (1) There are differences in the valence and arousal dimensions of basic musical emotional experiences between the two scale types, with music in major and minor scales inducing higher arousal levels than pentatonic music; there are also differences in the multidimensional aesthetic emotions between the two scale types; (2) Participants with varying levels of musical training experience may exhibit differences across multiple levels of musical emotional experiences; (3) The skin conductance and heart rate changes are greater for major and minor scales than for the pentatonic scale; (4) The skin temperature for the pentatonic scale is higher than for major and minor scales; (5) Musical training experience influences physiological indicators, with those participants having musical training experience showing higher physiological indicator values.

## 2 Methods

### 2.1 Participants

In this study, a total of 65 college student participants (48 females, M = 20 ± 1.34 years old) were recruited through campus advertisements and online announcements. They were divided into two groups: individuals with music training experience (34 participants, averaging over 5 years of music training, and concurrently undertaking vocal or instrumental training in both Chinese and Western styles) and individuals without music training experience (31 participants, no formal music training). All participants were right-handed, had no history of psychiatric disorders, and had normal hearing and vision (corrected). Before the experiment, all participants completed a demographic questionnaire, signed informed consent forms, and received compensation upon completion of the experiment. This study was approved by the Ethics Committee of Southwest University (Licence No. H23116).

### 2.2 Materials

This study plans to use Chinese traditional string solo pieces and Western string solo pieces as priming stimuli. Initially, 100 excerpts of Chinese traditional pentatonic music (duration 28–30s) were selected. Thirty-two music major college students rated the emotional valence and arousal of each mode on a scale of 1–9. The results showed significant differences in emotional valence between different modes of the Chinese traditional pentatonic scale, *F* (1,31) =6.83, *p* < 0.001, η^2^_p_ = 0.18, and significant differences in arousal, *F* (1,31) =33.67, *p* < 0.001, η^2^_p_ = 0.52 (see Table 2). Using 5 as the midpoint of the 9-point scale, no significant differences in emotional valence were found for the Shang, Jue, and Zhi modes (*all ps* > 0.05), only the Gong mode [*t*_Gong_(31) = 2.76, *p* = 0.010] and Yu mode [*t*_Yu_(31) = 3.533, *p* = 0.001] showed significant differences compared to other modes. Thus, based on the mean valence of the pentatonic modes, we selected ten Chinese traditional string solo pieces in the Yu and Shang modes, each lasting 30 s, and ten Western classical string solo pieces in major and minor keys, each lasting 30 s, to compare the induced emotional effects of the two modalities. To avoid differences in emotional expression between the two modalities’ musical materials, we selected five pieces each expressing happiness and sadness from both modalities. The paired-sample t-tests was conducted to verify that there were no significant differences in overall valence and arousal between the musical excerpts of the two modalities, [*t*_happy valence_(31) = 2.02, *p* = 0.051], [*t*_happy arousal_(31) = − 1.29, *p* = 0.206], [*t*_sad valence_(31) = − 1.29, p = 0.206],[*t*_sad arousal_(31) = 1.07, *p* = 0.289].

**Table 2:** The emotional valence of Chinese traditional music pentatonic scale (*n* = 32).

Mode	Gong	Shang	Jue	Zhi	Yu
	M ± SD	M ± SD	M ± SD	M ± SD	M ± SD
Valence	5.34 ± 0.71	4.97 ± 0.79	5.11 ± 0.92	5.10 ± 0.83	5.45 ± 0.73
Arousal	4.59 ± 0.94	3.50 ± 1.01	3.34 ± 1.03	4.27 ± 1.08	4.55 ± 0.93

### 2.3 Procedure

**Figure 2:**
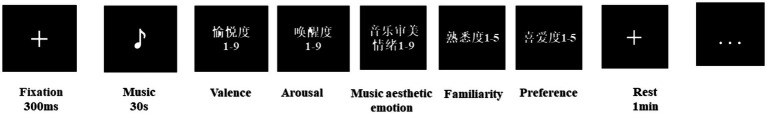
Emotional experience flowchart in music.

The study employed a passive listening paradigm, combining subjective reports with physiological measurements. Experimental procedure: Initially, a white crosshair appeared at the center of a black computer screen, prompting participants’ attention. Subsequently, a music excerpt (30s) was played. Following the music presentation, participants sequentially rated the self-induced pleasantness (1–9), arousal (1–9), nine aesthetic emotions (1–9), familiarity (1–5), and preference (1–5) of the music segment. To prevent participants from giving the same score repeatedly, familiarity and liking ratings used different scales from other dimensions. After each music segment, there was a 1-min rest period before proceeding to the next trial. The experiment comprised 2 blocks, totaling 20 trials. To prevent consecutive presentation of similar music, music stimuli were presented in a pseudo-randomized fashion (see Figure 2).

### 2.4 Experimental design

We adopted a 2 (Musical Scale: Chinese Traditional Pentatonic Scale, Western Diatonic Scale) × 2 (Music Training Type: Music Majors, Non-Music Majors) × 2 (Types of emotional expression in music: Happy, Sad) mixed experimental design, with music training type as a between-subjects factor and musical scale as a within-subjects factor.

### 2.5 Measurement tools

Subjective measures will collect the valence, arousal, familiarity, and liking of music excerpts in different scales, along with the assessment of aesthetic emotional intensity using the Geneva Emotion Music Scale (GEMS) (Zentner et al., 2008).

Physiological measures utilized the MP150 16-channel biofeedback system to record three physiological signals: skin conductance (SC), skin temperature, and heart rate. SC was recorded using electrodes attached to the fingertip of the left hand’s middle and ring fingers, while skin temperature was recorded using a thermistor connected to the index finger of the left hand. Heart rate was measured using a photosensitive sensor attached to the thumb of the left hand.

### 2.6 Statistical analysis

Following previous research (Bai et al., 2016), the mean values of SC, skin temperature, and heart rate in the 15 s preceding the onset of music playback in each block were used as the “baseline” levels for these physiological indicators. The mean values of the three physiological indicators for each mode of music were then subtracted from their respective “baseline” levels. Additionally, based on prior studies, the SC data were subjected to a Ln transformation. These resultant values of the three indicators were then imported into SPSS 27.0 (IBM, New York, NY, United States) software for analysis of variance (ANOVA) as measures of experimental effects.

## 3 Results

### 3.1 Behavioral analysis

A repeated measures analysis of variance was conducted with pleasure as the dependent variable. The main effect of music tonality type was significant *F* (1,63) =5.68, *p* = 0.020, η^2^_p_ = 0.08, indicating that pleasure ratings were higher for pentatonic music than for major and minor tonalities. Furthermore, a significant interaction was found between music tonality type and the expressed emotion of the music *F* (1,63) =36.10, *p* < 0.001, η^2^_p_ = 0.36. Simple effects revealed that pleasure ratings were significantly higher when listening to music expressing happiness compared to sadness; however, no significant differences were observed for music expressing sadness. There were no significant differences between groups.

Repeated measures analysis of variance was conducted with arousal as the dependent variable. The main effect of music tonality type was significant *F* (1,63) =25.51, *p* < 0.001, η^2^_p_ = 0.28, indicating higher arousal ratings for major and minor tonalities compared to pentatonic music. A significant interaction was found between music tonality type and the expressed emotion of the music *F* (1,63) =9.23, *p* = 0.003, η^2^_p_ = 0.12. When listening to music expressing happiness, arousal ratings were higher for Western tonalities compared to pentatonic music; similarly, when listening to music expressing sadness, arousal ratings for major and minor tonalities were higher than those for pentatonic music. Additionally, a significant interaction was found between music expression and participant type *F* (1,63) =7.25, *p* = 0.009, η^2^_p_ = 0.10. When listening to music expressing happiness, there were no significant differences in arousal ratings between the two participant groups; however, when listening to music expressing sadness, participants without music training experience rated arousal higher than music-trained participants. There were no significant differences between groups.

A repeated measures analysis of variance was conducted with familiarity as the dependent variable. The main effect of music tonality type was not significant *F* (1,63) =0.99, *p* = 0.323, but there was a significant interaction between music tonality type and participant type *F* (1,63) =9.10, *p* = 0.004, η^2^_p_ = 0.12. Music-trained participants rated higher familiarity when listening to Chinese traditional music compared to those without music training *F* (1,63) =9.61, *p* = 0.003, η^2^_p_ = 0.13, and similarly, when listening to Western tonalities, music-trained participants rated higher familiarity compared to those without music training *F* (1,63) =33.55, *p* < 0.001, η^2^_p_ = 0.34.

**Figure 3:**
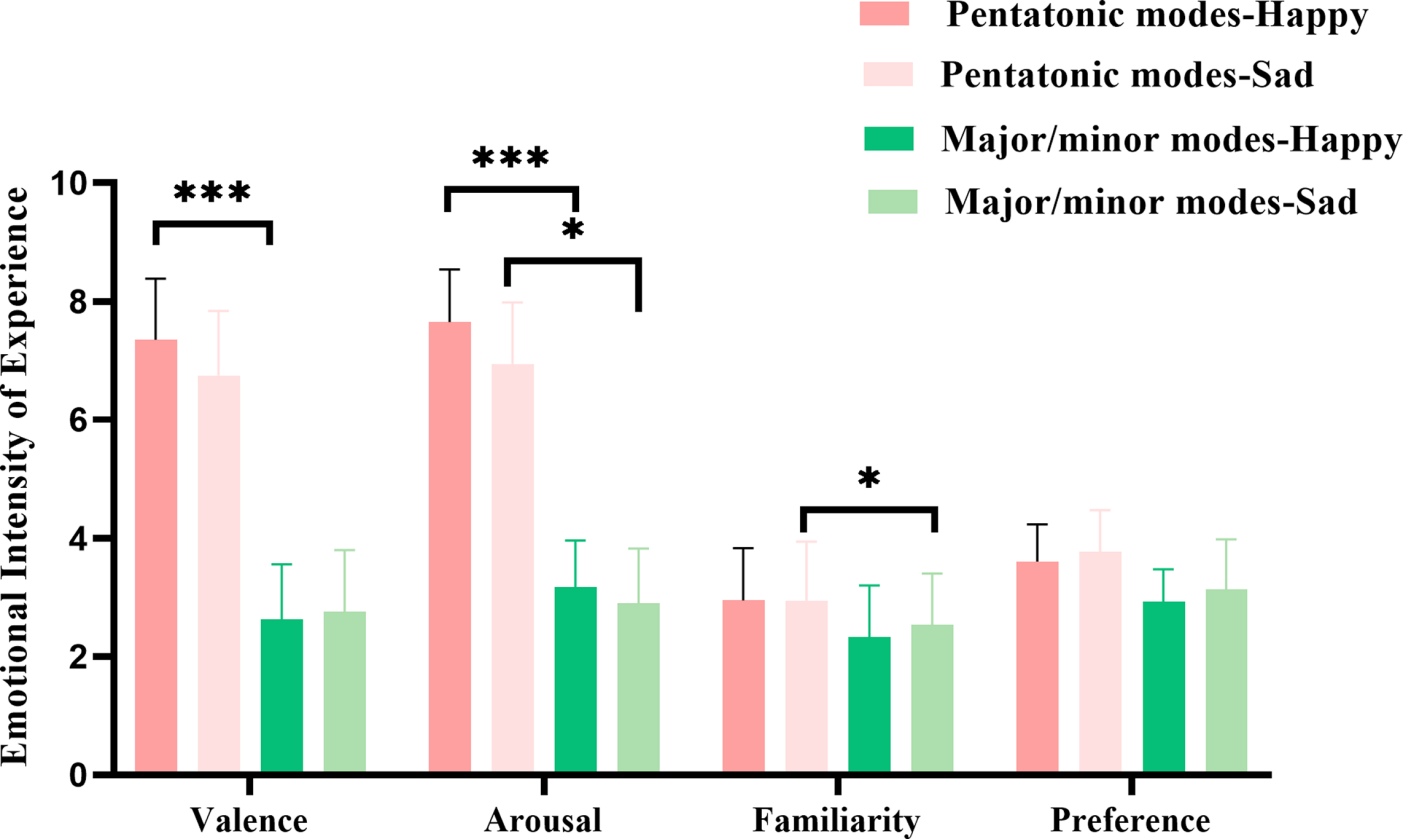
Differences in basic emotional valence, arousal, familiarity, and preference between chinese traditional pentatonic and western major/minor music: A graphical representation. *p <0.05, **p <0.01, ***p <0.001.

A repeated measures analysis of variance was conducted with liking as the dependent variable. The main effect of music tonality type was significant *F* (1,63) =5.51, *p* = 0.022, η^2^_p_ = 0.08, indicating higher liking ratings for Chinese traditional music compared to Western major and minor tonalities. There was also a significant interaction between music tonality type and participant type *F* (1,63) =8.98, *p* = 0.004, η^2^_p_ = 0.12. Music-trained participants showed higher liking ratings for Western music compared to those without music training, while no significant differences were observed when listening to Chinese traditional music. There were no significant main effects or interactions involving differences in music expression and other variables. Figure 3 illustrates the differences in basic emotional valence, arousal, familiarity, and liking ratings between Chinese traditional pentatonic and Western major and minor tonalities.

**Figure 4:**
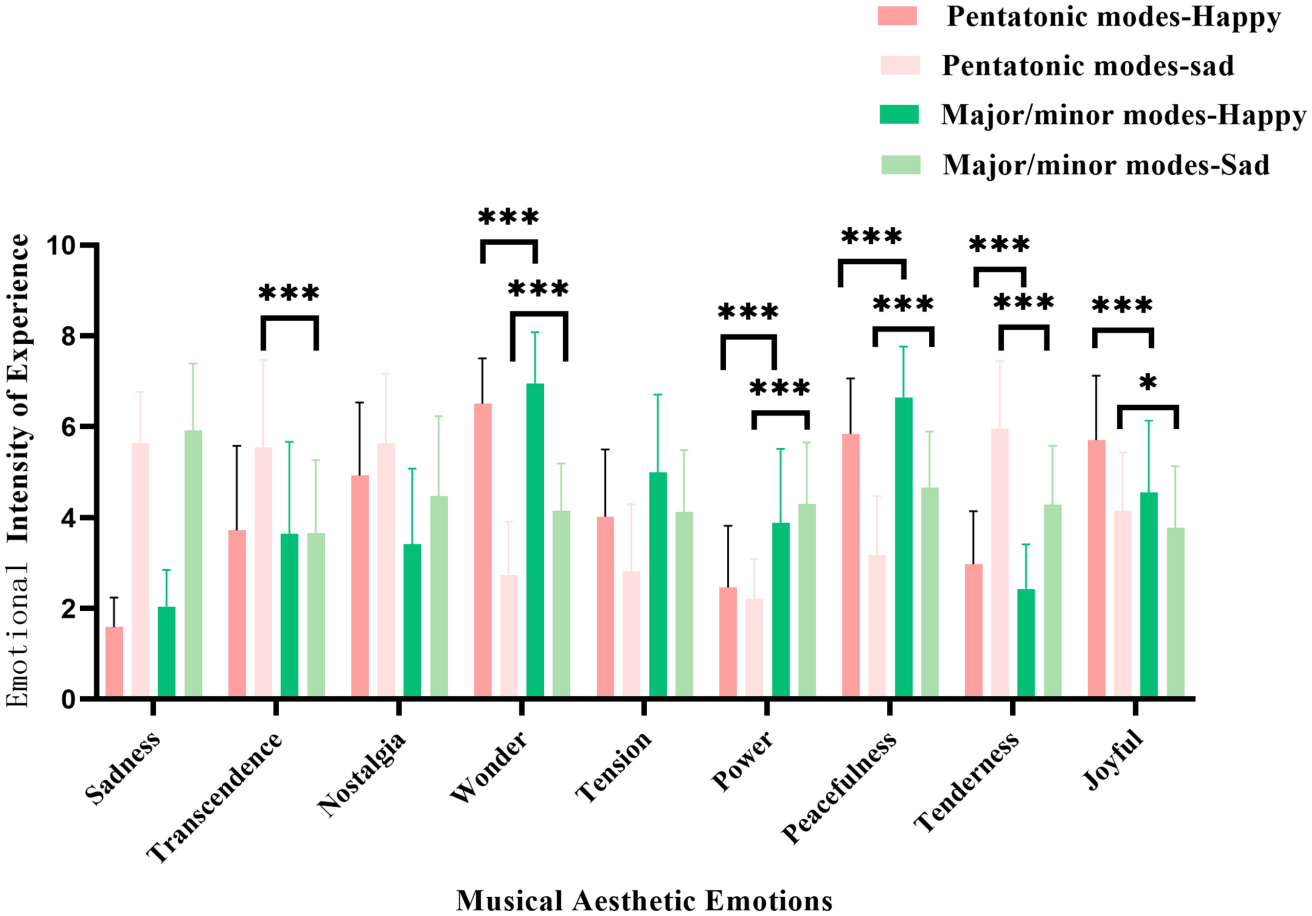
Differences in aesthetic emotional responses between chinese traditional pentatonic and western major/minor modes. *p <0.05, **p <0.01, ***p <0.001.

A repeated measures analysis of variance was conducted with aesthetic emotional experience as the dependent variable, the main effect of music scale was significant *F* (8,63) =5.50, *p* = 0.022, η^2^p = 0.08. Additionally, a significant main effect of music aesthetic emotion was found *F* (8,63) =33.53, *p* < 0.001, η^2^_p_ = 0.34, suggesting varied emotional responses across the aesthetic dimensions. Furthermore, a significant interaction effect between music scale and aesthetic emotion was observed *F* (8,63) =82.46, *p* < 0.001, η^2^_p_ = 0.56, indicating that the two musical scale modalities differed significantly across the nine aesthetic emotions (see Figure 4). Moreover, a significant interaction effect was found among music scale, emotional expression, aesthetic emotion, and participant type, *F* (8,63) =2.56, *p* = 0.009, η^2^_p_ = 0.39. Simple effects analysis revealed that participants with music training experience rated Western diatonic music higher than Chinese pentatonic music on aesthetic emotions of sadness, wonder, tension, power, and peacefulness while listening to joyful music. Conversely, they rated Chinese pentatonic music higher on aesthetic emotions of nostalgia, tenderness, and joyful but lower on sadness when listening to sad music. Participants without music training experience rated Chinese pentatonic music higher than Western diatonic music on aesthetic emotions of nostalgia, tenderness, and joyful but lower on sadness, tension, power, and peacefulness when listening to joyful music. Conversely, they rated major/minor diatonic music higher on aesthetic emotions of astonishment, tension, power, and peacefulness but lower on transcendence, nostalgia, and tenderness when listening to sad music.

### 3.2 Physiological results

Two participants had missing physiological data, leaving 63 participants’ data for analysis. Using t-tests, we compared the changes in skin conductance (SC), skin temperature, and heart rate between the two tonal modalities to baseline (0). For pentatonic music, significant differences were found in the change values compared to baseline:[*t*_Pentatonic/sc_ (62) = −2.91, *p* = 0.005], indicating a significant decrease in SC while listening to pentatonic music; [*t*_Pentatonic/ Temperature_ (62) = −74.11, *p* < 0.001], indicating a significant decrease in skin temperature; and [*t*_Pentatonic/Heart Rate_ (62) = −43.30, *p* < 0.001], indicating a significant decrease in heart rate. For major/minor music, significant differences were found in skin temperature and heart rate change values compared to baseline: [*t*_Major Minor/Temperature_ (62) = −62.21, *p* < 0.001], indicating a significant decrease in skin temperature; [*t*_Major Minor/Heart Rate_ (62) = −44.84, *p* < 0.001], indicating a significant decrease in heart rate. However, there was no significant difference in SC change values compared to baseline: t _Major Minor/sc_ (62) = −1.888, *p* = 0.064.

Using Ln skin conductance (SC) change values as the dependent variable, a repeated measures analysis of variance was conducted. The main effect of tonal modality was significant, *F* (1,61) =5.64, *p* = 0.021, η^2^_p_ = 0.08, indicating that pentatonic music elicited lower SC compared to major/minor music. The main effects and interaction effects of music expression, scale type, and participants were not significant. However, simple effects analysis revealed that when the expressed emotion was sadness, the electrodermal activity induced by Western diatonic music was greater than that induced by Chinese pentatonic music *F* (1,61) =4.96, *p* = 0.030, η^2^_p_ = 0.07, while differences were not significant for joyful music. Additionally, participants with music training experience showed greater electrodermal activity when listening to major/minor music compared to pentatonic music *F* (1,61) =5.85, *p* = 0.018, η^2^_p_ = 0.08, with no significant differences among non-music participants.

Using skin temperature change values as the dependent variable, a repeated measures analysis of variance was conducted. The main effect of tonal modality was significant *F* (1,61) =6.99, *p* = 0.010, η^2^_p_ = 0.10, indicating that pentatonic music elicited higher skin temperature compared to major/minor music. The interaction effect between music expression of emotion and music scale type was significant *F* (1,61) =4.49, *p* = 0.038, η^2^_p_ = 0.16. Simple effects analysis revealed that when the expressed emotion in music was sadness, individuals exhibited greater changes in skin temperature in response to Chinese pentatonic music compared to diatonic music *F* (1,61) =7.60, *p* = 0.008, η^2^_p_ = 0.11. However, when the expressed emotion was happiness, the difference between the two was marginally significant *F* (1,61) =3.72, *p* = 0.058, η^2^_p_ = 0.05.

**Figure 5:**
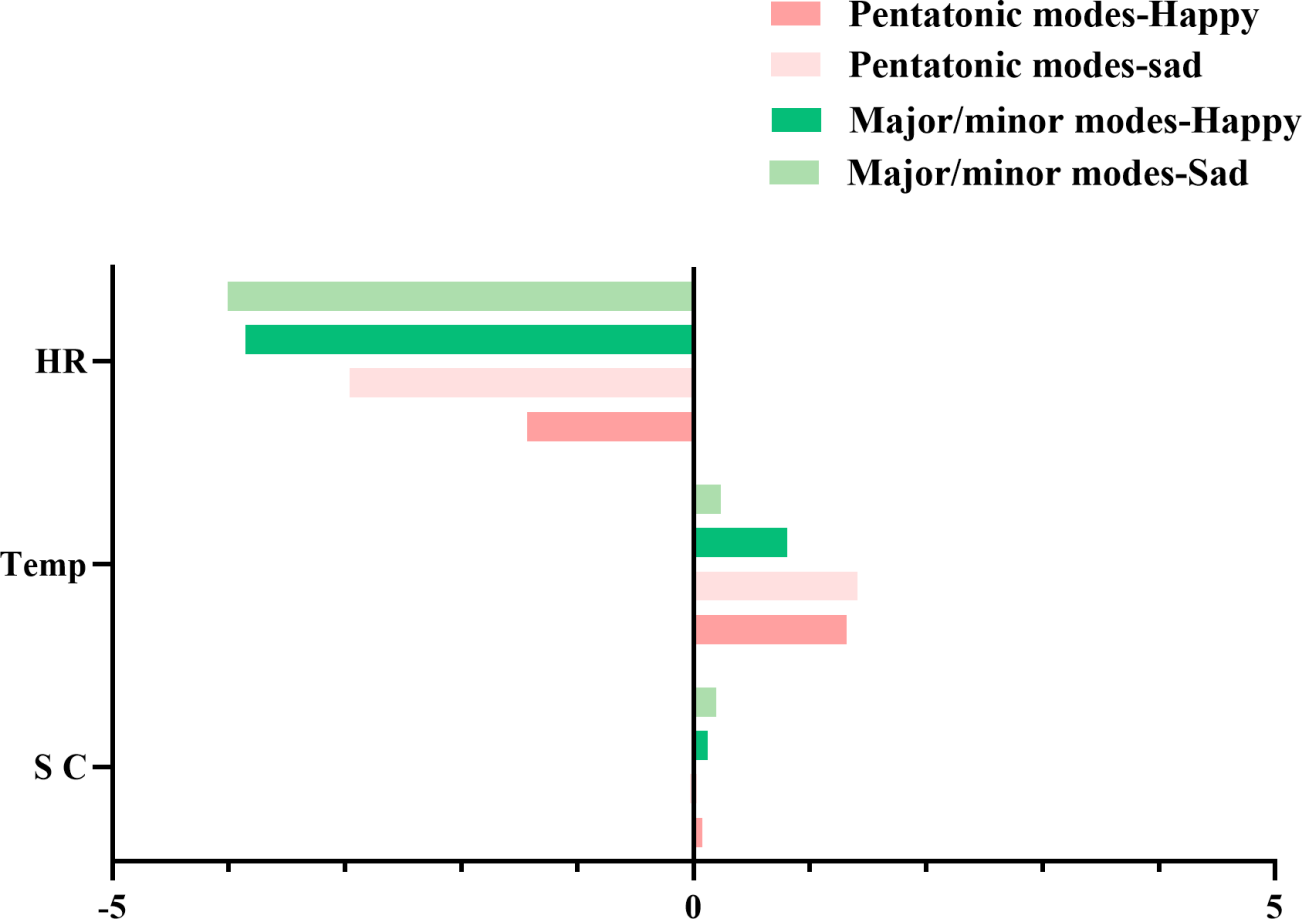
Contrasting physiological responses to traditional chinese pentatonic and western diatonic scales.

Using heart rate change values as the dependent variable, a repeated measures analysis of variance was conducted. The main effect of tonal modality was significant *F* (1,61) =4.53, *p* = 0.037, η^2^_p_ = 0.06, indicating that major/minor music elicited faster heart rates compared to pentatonic music. The main effects and interaction effects of music expression type, scale type, and participant type were not significant. However, simple effects analysis revealed that when the expressed emotion in music was sadness, individuals exhibited greater heart rate variability in response to pentatonic music compared to diatonic music *F* (1,61) =9.28, *p* = 0.003, η^2^_p_ = 0.13, whereas no significant difference was found when the expressed emotion was happiness. Figure 5 illustrates the contrast in physiological responses of participants to pentatonic and major/minor scales.

## 4 Discussion

The hidden structures within each pitch determine the psychological resonance we experience with different modes, which is the primary reason for the rich emotional experiences brought by different modes (Pu, 2021). The differences in mode formations between Chinese and Western music are influenced by both external cultural factors and internal mechanisms. Externally, Chinese and Western music have different cultural backgrounds and aesthetic pursuits. Traditional Chinese music excels in expressing the aesthetic art of harmonizing life and nature, where music originates from life and serves life. On the other hand, Western music is less influenced by external environmental factors, built upon a rigorous logical foundation, and emphasizes the professionalism of musical art. The internal mechanisms influencing the differences in mode formations between Chinese and Western music lie in the tension differences within the mode structures. The internal energy of the major and minor modes, based on the fifth-degree chain structure, is equal to the sum of the Gong, Shang, Jue, Zhi, and Yu modes. Therefore, each mode averages a weaker energy compared to major and minor modes. The pentatonic system revolves around the fifth-degree chain, which exerts significant control, making it closer to the fifth-degree chain structure and thus weakening the mode’s construction power. In contrast, Western major and minor modes, deviating from the fifth-degree chain center, experience less control, allowing them to distance themselves from the fifth-degree chain structure and exhibit strong mode construction power (Pu, 2020). The differences in mode construction power between Chinese and Western modes determine the differences in melodic forms, influencing the emotional experiences of listeners.

The findings of this study largely support the hypothesis. Behavioral results indicate that individuals rated Chinese pentatonic music higher in basic emotional pleasantness compared to major/minor tonalities, suggesting that pentatonic music may elicit more positive emotional experiences. Additionally, individuals exhibited higher arousal levels towards major/minor tonalities, which may be attributed to the differing tonal construction strengths between the two systems - major/minor tonalities having stronger construction strength compared to pentatonic tonalities, influencing the arousal level of music emotions. While familiarity did not show a significant main effect on tonality types, musical training experience facilitated familiarity in music emotion experiences, with trained participants demonstrating greater familiarity towards pentatonic music. Moreover, individuals expressed greater preference towards pentatonic music, particularly those with musical training experience, indicating a close relationship between aesthetic judgment and motivation towards aesthetic approach or avoidance, thereby determining individual preferences in music aesthetics. Accumulating rich experiences through long-term musical training enhances familiarity and preference towards traditional music. Research indicates that audiences without relevant musical experience can still experience emotions, but they are influenced by their music cultural experience (Pereira et al., 2011), Brattico et al. (2016) found that participants with music training experience exhibited stronger activation in the anterior insula and the cingulate area when expressing preferences for music compared to those without music training experience. Within the BRECVEMA model framework, three mechanisms—brain stem reflexes, rhythmic entrainment, and emotional contagion—are closely linked to the body’s natural reactions, showing a lower dependence on music cultural experience. Conversely, five mechanisms—evaluative conditioning, visual imagery, episodic memory, musical expectancy, and aesthetic judgment—exhibit a higher dependence on music cultural experience (Juslin and Västfjäll, 2008; Juslin, 2013). The Chinese university students selected for this study were simultaneously exposed to both Chinese and Western music. However, they consistently demonstrated a higher level of familiarity with and preference for traditional Chinese music. This indicates that both music training experience and the individual’s music cultural background collectively contribute to their preference for Chinese traditional music culture. In the aesthetic emotion evaluation process, pentatonic and major/minor tonalities exhibit significant differences across various aesthetic emotion types. Pentatonic tonalities evoke significantly stronger emotional experiences in transcendence, nostalgia, serenity, warmth, and joy, while major/minor tonalities elicit stronger emotional experiences in sadness, awe, tension, and power. The Geneva Emotion Music Scale (GEMS) categorizes nine aesthetic emotions into low, medium, and high arousal dimensions (Zentner et al., 2008), suggesting differences in arousal levels across aesthetic emotions between the two tonality types.

The arousal level of music emotion is a key factor influencing the level of physiological indicators (Juslin and Lindström, 2010; van der Zwaag et al., 2011; Bai et al., 2016). In this study, compared with baseline 0, skin temperature and heart rate decrease after listening to music. In terms of electrodermal indicators, the electrodermal indicators of pentatonic music decrease, and although there is no significant difference in the electrodermal indicators of major and minor mode music, the mean electrodermal changes are lower than baseline, indicating that the individual’s autonomic nervous system is influenced when listening to music, leading to a decrease in sympathetic nervous activity and an increase in parasympathetic nervous activity, resulting in changes in physiological indicators such as skin conductance, skin temperature, and heart rate, which can affect individual emotions, making individuals feel relaxed and calm (Juslin and Västfjäll, 2008; Coutinho and Cangelosi, 2011). Previous explorations of physiological indicators have yielded inconsistent results, with some studies indicating that minor mode music has a higher electrodermal increase rate than major mode music (Nater et al., 2006), while others believe that the electrodermal increase rate of major mode music is higher than that of minor mode music (Lundqvist et al., 2009), and some studies have found no effect of mode on electrodermal changes (Bai et al., 2016). Therefore, electrodermal indicators are directly related to the subjective arousal level of emotions (Levenson, 2014; Bai et al., 2016), and the results of this study are consistent with previous research results, namely, the electrodermal increase rate induced by pentatonic mode is lower than that of major and minor modes, which is also consistent with behavioral results, indicating that the emotional arousal level of major and minor modes is higher. With the increase of emotional arousal level, sympathetic nervous activity is enhanced, resulting in a decrease in skin resistance and an increase in electrodermal value. Meanwhile, participants with music training experience have a higher electrodermal change rate than those without music training experience, and participants with music training experience have a higher electrodermal change rate for major and minor modes than for pentatonic mode, which indicates that music training has an impact on the electrodermal physiological indicators of emotional experiences, and participants with music training experience consider major and minor modes to have a stronger arousal experience. In terms of skin temperature change indicators, previous studies have shown that skin temperature change values are correlated with emotional valence (Kreibig, 2010; Levenson, 2014; Bai et al., 2016). In this study, the amplitude of skin temperature change in pentatonic mode is greater than that in major and minor modes, which is consistent with behavioral results, namely, the emotional valence of pentatonic mode is greater than that of major and minor modes, and higher emotional valence induces larger skin temperature change indicators. In terms of heart rate change indicators, according to previous research results, heart rate more represents the intensity of emotional arousal (Pfister et al., 2011; Levenson, 2014; Bai et al., 2016). Consistent with previous research results, in this study, the emotional arousal level of Western major and minor modes is greater than that of pentatonic mode, and heart rate change indicators are consistent with physiological results. Furthermore, we also found that the expressed emotion in music has a certain influence on emotional experiences. A study on the automatic attentional capture of emotional and non-emotional visual stimuli indicated that the intensity, latency, and location of neural activity associated with automatic attention are significantly related to the emotional content of the stimuli (Carretié et al., 2004). Our research corroborates this viewpoint, and we further discovered that the physiological responses of participants (skin conductance, skin temperature, and heart rate) differed predominantly during music evoking sadness. This may stem from the preference of college students for sad emotional music in Chinese traditional music (Liu et al., 2022), or perhaps because sad music elicits emotional resonance in participants, leading to increased physiological responses.

There are some limitations in this study. On the one hand, the participants chosen for this study were university students with a Chinese cultural background, who may be more familiar with and inclined towards traditional pentatonic music due to their exposure to traditional cultural environments. Future research could involve participants from other cultural backgrounds for cross-cultural comparative studies. On the other hand, this study only considered the participants’ musical training background, but there are many other individual factors that can influence emotional experiences, such as gender and personality traits. Therefore, in subsequent studies, further control or exploration of different variables can be conducted.

In summary, from a physiological perspective, listening to music can effectively improve individuals’ emotions. Furthermore, combining behavioral and physiological indicators reveals that individual changes in skin conductance and heart rate are associated with the level of emotional arousal induced by music, while changes in skin temperature reflect the emotional valence of the music. The magnitude of tension in the construction of musical modes determines the intensity of emotional arousal in music. In this experiment, by comparing the differences in basic emotions and aesthetic emotional intensity between pentatonic and major/minor mode music, it was found that Chinese university students perceive traditional Chinese pentatonic music to be more pleasant and enjoyable, while considering major/minor mode music to have higher arousal levels. This aligns with our theoretical assumptions and physiological indicators. In terms of aesthetic emotions, significant differences were observed in various dimensions between the two mode types, indicating that the emotional induction effects of the two types of music differ in the process of aesthetic emotional experience. Therefore, the results of this study confirm the theory of cultural specificity, suggesting that in the process of music emotional experience, both traditional Chinese music and Western classical music possess their own cultural schemas. Moreover, Chinese university students, especially those with music training experience who have been exposed to the traditional Chinese cultural environment for a long time, have accumulated a wealth of implicit experiences in music culture, showing a bias towards familiarity and preference for traditional Chinese music compared to Western classical music. This study not only explains the emotional induction effects of mode types on music emotional experience from a physiological perspective, providing empirical evidence for cross-cultural and localized research on music emotion, but also offers a certain reference basis for the application of music in health care.

## 5 Conclusion

Music, as the primary channel for human emotional communication, can influence emotions both through natural reactions of the body and through the individual’s music cultural experience and cognitive construction. Research findings indicate that the construction intensity of musical modes is a significant factor affecting emotional experiences and physiological changes. Skin conductance and heart rate changes are relevant indicators reflecting the arousal level induced by music, with Western major-minor mode music inducing higher arousal, thus resulting in greater skin conductance and heart rate changes compared to pentatonic mode music. Skin temperature changes serve as effective indicators of emotional valence, with participants in this study perceiving higher levels of pleasantness in pentatonic mode, consequently exhibiting higher skin temperature changes. Additionally, the results suggest that music training experience can facilitate individuals’ familiarity with and preference for pentatonic mode.

Music cultural experience influences an individual’s perception and understanding of music, determining their preferences and responses to different musical forms, additionally, it reflects diverse cultural interpretations and expressions of music. This study employed Chinese university students as experimental participants. Although unfamiliar music pieces were chosen for the experiment, participants’ music cultural experience may still affect their music emotional experiences and physiological indicators. Therefore, future research should further explore the emotional induction effects of Eastern and Western mode music on participants from different cultural backgrounds. By comparing music cultural experiences across different cultures, a deeper understanding of diverse cultural attitudes, values, and aesthetic standards regarding music can be achieved, thereby promoting cross-cultural communication and understanding.

## Data availability statement

The original contributions presented in the study are included in the article/supplementary material, further inquiries can be directed to the corresponding author.

## Ethics statement

The studies involving humans were approved by the Ethics Committee of Southwest University. The studies were conducted in accordance with the local legislation and institutional requirements. The participants provided their written informed consent to participate in this study.

## Author contributions

YJ: Conceptualization, Methodology, Software, Validation, Formal analysis, investigation, Data curation, Writing —original draft preparation, Writing — review & editing, Visualization, Project administration. MZ: Conceptualization, Resources, Writing — review & editing, Supervision, Project administration, Funding acquisition.
